# Single-Atom Iron Catalysts with Core-Shell Structure for Peroxymonosulfate Oxidation

**DOI:** 10.3390/molecules29153508

**Published:** 2024-07-26

**Authors:** Jielei Fan, Ruoxue Wang, Xiaodong Zheng, Hancheng Jiang, Xiuli Hu

**Affiliations:** Institute of Polymer Science and Engineering, School of Chemical Engineering, Hebei University of Technology, Tianjin 300130, China

**Keywords:** covalent organic framework, peroxymonosulfate, single-atom iron catalyst, porous material, water pollution

## Abstract

The chemical tolerance of ketoenamine covalent organic frameworks (COFs) is excellent; however, the tight crystal structure and low surface area limit their applications in the field of catalysis. In this work, a porous single-atom iron catalyst (FeSAC) with a core–shell structure and high surface area was synthesized by using Schiff base COF nanospheres as the core and ketoenamine COF nanosheets growth on the surfaces. Surface defects were created using sodium cyanoborohydride etching treatment to increase specific surface area. The dye degradation experiments by peroxymonosulfate (PMS) catalyzed by the FeSAC proved that methylene blue can be degraded with a degradation rate constant of 0.125 min^−1^ under the conditions of 0.1 g L^−1^ catalyst dosage and 0.05 g L^−1^ peroxymonosulfate. The FeSAC/PMS system effectively degrades various pollutants in the pH range of 4–10 with over 80% efficiency for four cycles and can be recovered by soaking in iron salt solution. Free radical quenching experiments confirmed that singlet oxygen and superoxide radicals are the main active species for catalysis.

## 1. Introduction

In recent years, organic pollutants, such as dyes, antibiotics, and pesticides, have posed significant threats to the promotion of sustainable development in the ecological environment due to their difficult degradation characteristics and ecological hazards. Therefore, water purification technology, especially the treatment of organic pollutants, has become a hot topic in environmental research. Current methods for treating water pollutants are generally divided into three categories: (1) physical methods, including coagulation [1], adsorption [2,3] and membrane separation [4,5,6]; (2) chemical methods, including Fenton oxidation, ozonation, photocatalysis, and electrocatalysis; and (3) biological methods [7]. Among these, chemical methods, especially advanced oxidation processes (AOPs), have emerged as an effective means for degrading organic pollutants via the generation of reactive oxygen species (ROS) [8,9,10]. PMS has been widely used in AOPs due to its stability, wide pH range applicability, and strong degradation capabilities [11,12,13]. The generated multiple ROS (·OH, SO_4_^−^·, O_2_^−^·, and ^1^O_2_) by activated PMS can thoroughly degrade organic pollutants into water and carbon dioxide. So, effective transition metal catalysts that activate PMS under mild conditions are highly desirable. Shao et al. exploited the lattice oxygen/interstitial oxygen in layered perovskite oxide to catalyze the production of 1O2 through PMS, effectively eliminating phenol [14,15].

Traditional metal catalysts composed of various nano or micrometer-scale metal materials have been widely used in various fields, including chemical synthesis, water pollution purification, biomedicine, etc. However, due to fact that the catalytic reactions mainly occur on the specific reachable surfaces, the catalytic efficiency and selectivity of heterogeneous catalysts are relatively lower than homogeneous catalysts. For heterogeneous catalysts, surface metal atoms act as active sites in the catalytic process. The specific surface area of nanoparticles significantly increases with the decrease in nanoparticle size, accompanied by changes in the metal atom coordination environment, such as surface defects, surface atomic structure, and electronic structure, which significantly enhance the catalytic performance of the catalysts. In 2011, Zhang and others discovered that a single Pt atom anchored on FeO_x_ exhibited effect active sites for CO oxidation and their catalytic performance was three times higher than that of bulk Pt, and the concept of single-atom catalysts (SACs) was proposed [16]. SACs are heterogeneous catalysts where catalytic sites exist in the form of isolated single-metal atoms. Compared to traditional heterogeneous catalysts, SACs have outstanding advantages: (1) high selectivity and activity due to their unique electronic and physicochemical structures; (2) low metal usage, low cost, strong chemical activity, and low metal loss rate; and (3) clear chemical structure, stable synthesis process, and easy analysis of reaction mechanisms. SACs solved the dilemma between homogeneous catalysts with definite molecular structures and heterogeneous catalysts with high selectivity. SACs have been extensively applied in various fields, such as photocatalysis [17], electrocatalysis [18], biomedicine [19], and PMS catalysis [20,21,22].

COFs have a large specific surface area and uniform pore distribution, facilitating the high loading of metal atoms. At the same time, the substrate is easily adsorbed to metal sites to participate in reactions. The inner core of COF pores can be functionalized to enhance the interaction between metals and substrates and prevent detachment. In addition, the framework itself has an excellent adjustable electronic structure, which can synergistically catalyze metal sites. Meanwhile, COFs have good dispersibility and are easy to recover, making them excellent carriers for single atoms. However, due to the dependence of unstable reversible covalent bonds on COF formation, COFs with excellent morphology often have poor oxidation stability. Balancing chemical stability and excellent morphology is challenging. Wang et al. loaded iron elements onto the vacancies of ketoenamine COFs at low temperatures and prepared FeSAC@COF and NiSAC@COF [23]. The obtained two types of SACs@COFs have a pore structure, low metal loss rate, and excellent cyclic performance. Yao et al. thermally decomposed iron-loaded COF into carbides and utilized the crystal structure of COF to enhance the site distribution of single-atom catalysts [24]. They also constructed crystal defects through pyrolysis to enhance the catalytic activity, which was able to degrade 98% of methyl orange over a wide pH range (4.58–10.01) for 15 min.

**Scheme 1:**
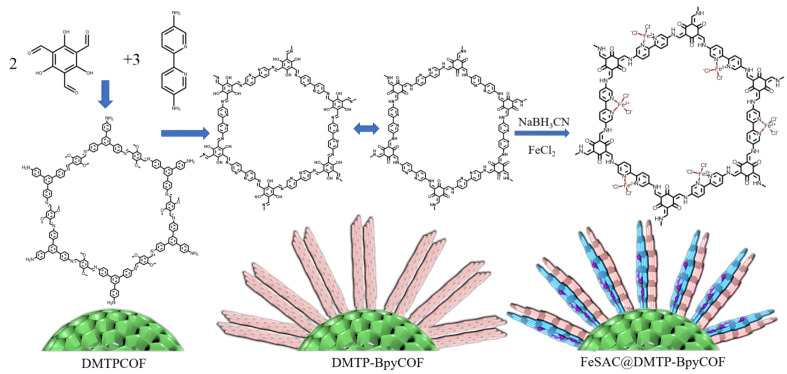
Schematic illustration of synthetic procedure of FeSAC@DMTP-BpyCOF.

In this study, a Schiff base COF (DMTPCOF, Figure S1) based on 2,5-dimethoxyterephthalaldehyde (DMTP) and 1,3,5-Tris (4-aminophenyl) benzene (TAB) was prepared. Then, ketoenamine COF (BpyCOF, Figure S2) sheets grew loosely and stacked on the DMTP core to form core–shell-structure DMTP-BpyCOF with uniformly dispersed DMTPCOF nanospheres as the core and BpyCOF sheets as the shell. Surface defects were introduced by chemical etching with sodium cyanide borohydride to increase the specific surface area, and Fe^2+^ was immobilized on Bpy to construct the FeSAC@DMTP-BpyCOF (Scheme 1). Chemical etching results in a large number of surface defects and pores in the stable and porous ketene amine COF, enhancing its ability to load metal elements. The loaded Fe can promote the transformation between Fe(III) and Fe(II), endowing the composite with excellent catalytic degradation performance [25]. The structure and properties of the material were characterized by XRD, FT-IR, UV-vis, SEM, HRTEM, and BET. Methylene blue (MB) was selected as a model pollutant to evaluate its catalytic activity. Cyclic experiments and ion interference experiments were conducted to evaluate the practicality of the materials. Free radical quenching experiments were used to investigate the catalytic mechanism.

## 2. Results and Discussion

### 2.1. Characterization of FeSAC@DMTP-BpyCOF

**Figure 1:**
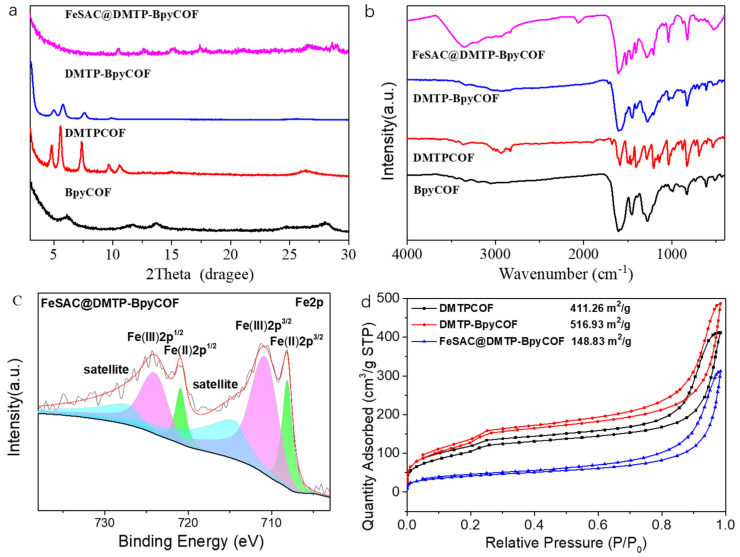
(**a**) XRD profiles of FeSAC@DMTP-BpyCOF and its precursors; (**b**) FT-IR spectra of FeSAC@DMTP-BpyCOF and its precursors; (**c**) Fe2p XPS profiles of FeSAC@DMTP-BpyCOF; (**d**) BET of FeSAC@DMTP-BpyCOF and its precursors.

The crystalline phases of FeSAC@DMTP-BpyCOF and its precursors were characterized by XRD as shown in Figure 1a. The peaks at 4.8°, 5.8°, 7.5°, and 26.3° in the XRD pattern of DMTPCOF and DMTP-BpyCOF correspond to (100), (110), (200), and (001) of DMTPCOF, respectively [26]. The peaks at 6.1°, 11.6°, 13.6°, and 28.0° correspond to (100), (110), (200), and (001) of BpyCOF, respectively [27]. FeSAC@DMTP-BpyCOF lattice distortion occurred after etching. We refer to the literature to speculate that it is in a 6-^i^Pr topology with AB stacking [28]. Its 10.4°, 12.6°, 15.1°, 17.4°, 26.7°, and 28.6° peaks may correspond to the (110), (200), (001), (101), (120), and (111) crystal plane. For DMTP-BpyCOF, only the DMTP characteristic peaks can be seen, indicating that the crystallinity of the DMTPCOF core is high while that of the Bpy shell is poor, resulting in weak diffraction in the XRD spectra. For FeSAC@DMTP-BpyCOF, which underwent chemical etching treatment, the characteristic peaks of DMTP significantly weakened, indicating that DMTPCOF is not resistant to reduction by sodium borohydride cyanide and crystal defects are formed.

FT-IR is employed to analyze the functional groups in the samples (Figure 1b). The BpyCOF exhibits peaks at 2800–3100 cm^−1^ (N-H stretching vibration peak), indicating the successful synthesis of DMTP-BpyCOF [29]. The strong N-H vibrational broad peak at 2800–3600 cm^−^¹ confirms the successful reduction of DMTP-BpyCOF by sodium cyanoborohydride and iron coordination to obtain FeSAC@DMTP-BpyCOF [27]. The presence of the C≡N vibrational peak at 2100 cm^−^¹ indicates that the cyanide groups generated by sodium cyanoborohydride coordinate with iron atoms and remain within the COF framework [30]. The elemental valence states of FeSAC@DMTP-BpyCOF and its precursor are analyzed by XPS, as shown in Figure S3. The area ratio of the pyridine N peak with a binding energy of 398.7 eV to the C-N-C peak with a binding energy of 400.0 eV in the N_1s_ spectrum is not 1:1, with a lower π-π* satellite peak, indicating the successful introduction of ketoenamine structure into the catalyst. The C=O characteristic peak with a binding energy of 288.2 eV appears in the C1s spectrum, and there are C=C-C=O characteristic peaks with a binding energy of 530.7 eV in the O1s spectrum [23], indicating that BpyCOF grows smoothly on the surface of DMTPCOF. In Figure 1c, the Fe2p spectrum shows a double peak of Fe(III) with a binding energy of 711.2 eV and a double peak of Fe(II) with a binding energy of 708.2 eV, indicating that Fe atoms are successfully loaded onto the surface of DMTP-BpyCOF; these satellite peaks originate from Fe2p_1/2_ and 2p_3/2_ ionization and shake excitations in the valence space mix strongly [31]. Due to the high porosity and deep loading depth of Fe atoms on the surface of DMTP-BpyCOF, 49.37% of Fe remains divalent and is not oxidized by oxygen in the air. The mixed-valence iron element can promote the decomposition cycle of PMS [25,32]. The surface loading rate of Fe element is quantitatively analyzed by XPS with a loading content of 2.13 wt%, indicating the enrichment of Fe on the catalyst surface.

The specific surface area and porosity analyzer was detected using BET, as shown in Figure 1d. The specific surface area of DMTPCOF is relatively large (411.26 m^2^/g). After modification with BpyCOF, the specific surface area of DMTP-BpyCOF further increases (516.93 m^2^/g), indicating that the core–shell structure can effectively increase the exposure area of DMTPCOF, thus providing more coordination sites on the surface. Sodium cyanoborohydride exhibits different etching effects on the two components of this catalyst. For the DMTPCOF core, which has a high specific surface area but low stability, sodium cyanoborohydride induces a strong etching effect, significantly reducing its crystallinity and specific surface area. In contrast, for the BpyCOF shell, which has a low specific surface area but high stability (Figure S4), sodium cyanoborohydride mainly etches some of the Schiff base conformations, resulting in an increase in the specific surface area and the number of surface defects. The overall specific surface area of the catalyst decreases. However, since iron atoms are primarily loaded onto the BpyCOF shell, the loading rate of iron atoms is higher in the etched catalyst. After etching and coordination with iron atoms, FeSAC@DMTP-BpyCOF maintains a specific surface area of 148.83 m^2^/g, which is higher than that of BpyCOF (61.47 m^2^/g). All these data confirmed that iron elements were successfully dispersed in single-atom form on the surface of FeSAC@DMTP-BpyCOF with uniform and porous structures.

The thermal stability of FeSAC@DMTP-BpyCOF and its precursors’ stability were analyzed by a Synchronous thermal analyzer (Figure S5). DMTPCOF exhibits strong thermal stability, remaining stable below 375 °C. BpyCOF, with lower crystallinity and containing a large number of residual hydroxyl and amino groups, undergoes dehydration and condensation below 155 °C and starts to decompose above 235 °C. FeSAC@DMTP-BpyCOF also shows dehydration weight loss below 125 °C, but from 125 °C onwards, its mass continuously decreases without a stable platform [33]. This is attributed to the Fe atoms activating the hydroxyl groups in the COF framework, generating hydroxyl radicals that attack the COF structure. However, its residual mass of 40.7% is not significantly lower than that of DMTP-BpyCOF, indicating that even under Fe-catalyzed degradation, the COF framework can maintain a certain level of stability, preserving the overall structural integrity of the catalyst.

### 2.2. Morphology Characterization

**Figure 2:**
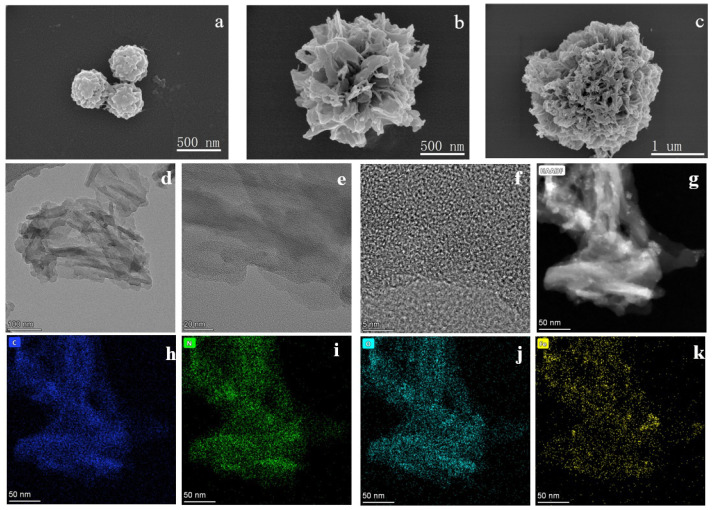
SEM images of (**a**) DMTPCOF, (**b**) DMTP-BpyCOF, (**c**) FeSAC@DMTP-BpyCOF; (**d**–**f**) HRTEM images of FeSAC@DMTP-BpyCOF; (**g**–**k**) EDS elemental mapping of FeSAC@DMTP-BpyCOF.

The morphology of FeSAC@DMTP-BpyCOF and its precursors DMTPCOF and DMTP-BpyCOF were characterized by SEM, TEM, and EDS, as shown in Figure 2. The SEM images show that DMTPCOF is a highly dispersed nanosphere with a diameter of 350–500 nm, and DMTP-BpyCOF presents a porous core–shell microsphere structure (1.1–1.3 μm) with DMTPCOF as the core and BpyCOF sheets growing loosely and stacking on the DMTPCOF core; the sheets’ stacking pattern is significantly different from the close packing of BpyCOF (Figure S6). On the surface of the DMTP-BpyCOF microspheres, many interlayer voids can be observed. Compared with DMTPCOF, the particle size significantly increased and the volume increment (>1000%) is significantly higher than the weight increment (120%), indicating that the Bpy shell has a very low packing density and extremely high porosity. For FeSAC@DMTP-BpyCOF, some layers collapsed and melted into spider-like structures due to a certain degree of chemical etching (Figure 2c). The spherical shape and high porosity were effectively preserved and the dispersibility did not show significant changes, demonstrating the successful synthesis of highly dispersed spherical porous FeSAC@DMTP-BpyCOF.

EDS analysis showed that C, N, O and Fe atoms were uniformly dispersed without significant aggregation. Further analysis of the microstructure of FeSAC@DMTP-BpyCOF by HRTEM revealed no large-scale striped crystal regions [34], while scattered diffraction patterns were visible, indicating a decrease in crystallinity after etching treatment and no stacking of metal atoms in the crystal structure. Moreover, many bright spots with a single-atom size can be clearly observed, confirming the uniform distribution of single-atom iron on the surface of DMTP-BpyCOF. In the BpyCOF shell, in addition to the bipyridine structure of Bpy that can load iron atoms, the ketoenamine structure of the COF can also load iron atoms [23]. The increased number of loading sites (Figure S7) enhances the iron atom loading rate and adds complexity to the catalytic mechanism.

### 2.3. Catalytic Activity

**Figure 3:**
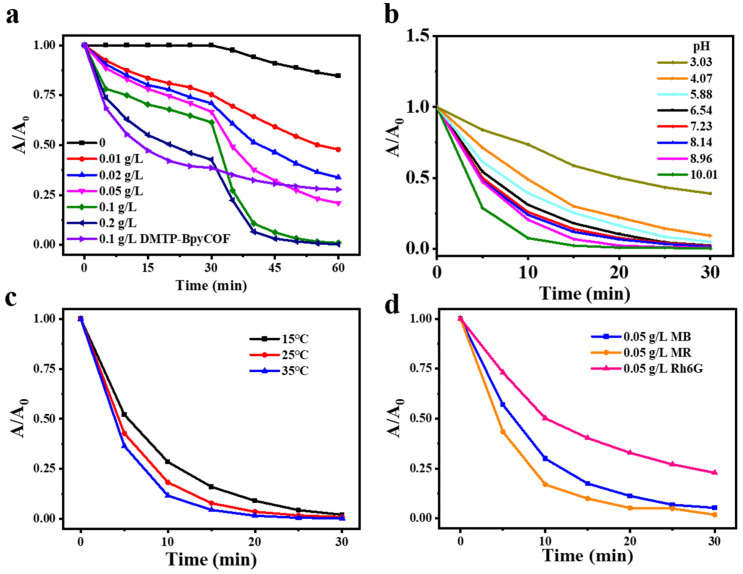
(**a**) Effect of the concentration of FeSAC@DMTP-BpyCOF on the degradation of MB ([PMS] = 0.1 g/L, [MB] = 0.05 g/L); (**b**) effect of pH value on catalytic efficiency ([PMS] = 0.05 g/L, [Mb] = 0.05 g/L, [Cat] = 0.1 g/L); (**c**) effect of temperature on degradation rate ([PMS] = 0.05 g/L, [Mb] = 0.05 g/L, [Cat] = 0.1 g/L); (**d**) the degradation rate of different pollutants ([PMS] = 0.05 g/L, [Cat] = 0.1 g/L).

The catalytic activity of FeSAC@DMTP-BpyCOF in activating PMS for the degradation of a model pollutant MB was investigated. The influences of catalyst dosage on the degradation rate were evaluated. To eliminate the interference caused by catalyst adsorption, a 30 min adsorption process was conducted prior to the addition of PMS (Figure 3a). The self-decomposition of PMS can slowly degrade MB, which is lower than 15%. The degradation rate significantly increases when the catalyst FeSAC@DMTP-BpyCOF is added. As the amount of catalyst increased, the degradation rate of MB accelerated (Table S1), indicating that FeSAC@DMTP-BpyCOF has catalytic activity towards PMS. At low concentrations, the catalyst can only catalyze the cracking of PMS to generate ·OH and SO_4_^−^·. As the catalyst concentration increases (>0.02 g/L), the instantaneous concentrations of ·OH and SO_4_^−^· in the system reach a threshold, which can react with PMS molecules to generate more active ^1^O_2_, which can completely degrade MB within 30 min under advanced oxidation [35]. DMTPBpyCOF without the iron element exhibits a significant and rapid adsorption effect due to its large specific surface area, while no catalytic effect on PMS is observed even at the concentration of 0.1 g/L. This indicates the efficient catalytic effect of single-atom iron-loaded FeSAC@DMTP-BpyCOF on the degradation of MB by PMS.

Considering the influence of PMS, catalyst, and dye concentration on the degradation effect, a series of experiments were conducted to determine the optimal degradation dosage as [PMS] = 0.05 g/L, [MB] = 0.05 g/L, [Cat] = 0.1 g/L (Figures S8 and S9). The effect of initial pH was evaluated by adjusting the pH value using NaOH and HCl (Figure 3b). The efficiency of the COF/PMS catalyst system in degrading pollutants increases with alkalinity due to the alkaline activation mechanism [12]. Under acidic conditions, the strong electrophilicity of hydrogen ions preferentially attacks negatively charged SO_4_^−^· and quenches it by binding with it, resulting in a significant inhibition of the degradation. At pH 4.13, the 60 min degradation rate is below 50%, and the catalytic activity of FeSAC@DMTP-BpyCOF is severely deactivated at pH < 3. The main active substance of PMS is KHSO_5_. HSO_5_^−^ exists in the form of high-activity SO_5_^2−^ when pH < 9.4 and in the form of low-activity SO_5_^2−^ when pH > 9.4 [36]. Therefore, PMS is not suitable when the pH is higher than 9.4. This system can maintain a degradation rate of over 90% for MB and good catalytic activity in the pH range of 5–10. This indicates that FeSAC@BpyCOF has an excellent pH applicability range.

The effect of temperature on the degradation efficiency is studied, and the results are shown in Figure 3c. The degradation rate increases with temperature. This suggests that temperature affects the degradation rate in two ways: (1) increasing the molecular diffusion rate, leading to faster adsorption and mass transfer processes; and (2) thermally activating PMS. The apparent activation energy calculated by the Arrhenius formula is 16.93 kJ/mol, which is lower than the peroxide bond energy in PMS and the activation energy under traditional heterogeneous metal catalysts [37]. This indicates that under the action of FeSAC@DMTP-BpyCOF, PMS bonds are highly activated, and the reaction rate is less affected by temperature. Thus, the reaction can proceed at lower room temperatures without additional heating. The ability of the catalyst to degrade different pollutants by PMS was studied to verify its universality, as shown in Figure 3d. Under catalytic conditions, PMS exhibits high degradation rates for MB, MR, and Rh6G. Over 90% of MB and MR can be degraded within 30 min. The degradation rate of Rh6G is slightly lower than that of MB and MR, with only 77.25% degradation within 30 min due to the abundant benzene ring structure and strong antioxidant properties of rhodamine dyes. These results show that FeSAC@DMTP-BpyCOF catalysts can efficiently activate PMS to degrade various organic pollutants.

**Figure 4:**
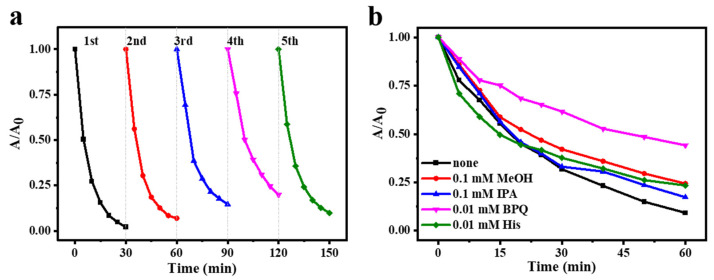
(**a**) Cyclic stability test of catalyst ([PMS] = 0.05 g/L, [Mb] = 0.05 g/L, [Cat] = 0.1 g/L); (**b**) effect of quencher on degradation rate ([PMS] = 0.02 g/L, [Cat] = 0.1 g/L, [Mb] = 0.05 g/L).

The cycling stability of the catalyst was studied in Figure 4a. After 30 min of degradation, the catalyst was centrifuged, washed with water, and heated for the next cycle. After four cycles, the metal sites were regenerated by soaking in a ferrous chloride solution to replenish the iron leaching. The degradation rate of MB by the catalyst COF/PMS reaches 97.89% within 30 min and maintains a high degradation rate of 93.06% in the second cycle. However, from the third cycle onwards, the degradation rate drops to below 90%, and in the fourth cycle, it barely maintains a degradation rate of 80.16%. After metal replenishment regeneration, the degradation rate of MB recovered to 91.53%, indicating that the activity loss of the catalyst is divided into two parts: a small amount of metal loss and pore collapse caused by COF oxidation and degradation. Metal loss is reversible and can be replenished, while pore collapse and quality loss are irreversible (Figure S10). The oxidation stability of FeSAC@DMTP-BpyCOF is significantly improved via etching treatment and exhibits a certain degree of cycling stability. However, it cannot be used for long-term cycling in strong oxidizing environments.

### 2.4. Possible Mechanism

To explore the degradation mechanism of MB, free radicals involved in the degradation reactions were distinguished through free radical quenching experiments as shown in Figure 4b. Isopropanol (IPA), methanol (MeOH), p-benzoquinone (BPQ), and L-histidine (His) were used as radical scavengers and added to the system. The degradation rate of MB decreased by 8.0%, 15.1%, 34.9%, and 14.1%, respectively, within 60 min, indicating that more than one active intermediate is involved in this catalytic reaction. Both IPA and MeOH can quench ·OH, while MeOH containing α-H can selectively quench SO_4_^−^. By comparing the quenching effects of IPA and MeOH, it can be inferred that the contributions of ·OH and SO_4_^−^· to the degradation rate are roughly equivalent and relatively small. BPQ induced the highest degradation rate decrease, which can selectively quench ^1^O_2_, indicating that ^1^O_2_ is the main active intermediate in this catalytic reaction. When ·O_2_^−^ was specifically quenched by His, a degradation rate decrease of 14.14% was observed, indicating that ·O_2_^−^ is another main active intermediate in this catalytic reaction [38]. Additionally, by using radical scavengers to directly capture ROS for electron paramagnetic resonance (EPR) testing, we directly observed the signals of ROS combined with the scavengers (Figure S11). In the aqueous phase, hydroxyl radicals combined with the DMPO scavenger exhibited a typical 1:2:2:1 quartet, accompanied by a sextet signal of peroxyl radicals combined with DMPO. In the methanol phase, superoxide radicals combined with DMPO displayed a fine-structured quartet with equal intensity. In the aqueous phase, singlet oxygen combined with TEMP showed an equal-intensity triplet signal [34]. These data confirm the presence of these reactive oxygen species during the degradation process. During the catalytic generation of ROS by PMS, ferrous iron is oxidized to ferric iron. The bipyridine structure retained in BpyCOF easily donates electrons to ferric iron, generating holes and ferrous iron. These holes can also activate PMS and pollutants, promoting degradation. However, due to the limited conjugation range on the BpyCOF surface, the transfer of holes that are confined to the COF surface is relatively slow, making it difficult to activate other molecules within their lifetime [39]. Consequently, their contribution to the degradation process is less significant than that of freely diffusing ROS, and the primary degradation mechanism relies on facilitating the redox cycling of iron [24].

To simulate the degradation environment in actual water bodies and verify the influence of active substance types, water interference tests are conducted by adding 0–40 mM chloride ions, bicarbonate ions, dihydrogen phosphate ions, and humic acid (HA). The results are shown in Figure S12. The inhibition of MB degradation rate by chloride ions is not very obvious at both 5 and 40 mM and chloride ions exhibit inhibition on MB degradation at 10 mL, which can be explained by the fact that chloride ions react with active free radicals ·OH and SO_4_^−^· to generate Cl·, which has a lower oxidation ability and slows down the degradation rate of MB. However, at high concentrations, Cl· can continue to attack PMS to regenerate ·OH and SO_4_^−^· or react with water to generate strong oxidizing HOCl to maintain the degradation capacity [40]. Dihydrogen phosphate has almost no inhibitory effect on the degradation, proving that FeSAC@DMTP-BpyCOF functions mainly as heterogeneous catalysis via iron fixed on the surface rather than leaching ions. The inhibition of the degradation rate by bicarbonate is extremely strong, which greatly blocks the continuation of the degradation reaction at 40 mM. This is because bicarbonate can react with all ROS to produce extremely low oxidizing CO_3_^−^, and even directly react with PMS [41]. HA has a certain inhibitory effect on the catalytic system, mainly reducing the degradation rate of MB in the form of competitive degradation. In summary, the possible catalytic mechanisms of FeSAC@DMTP-BpyCOF/PMS system is summarized as follows:

(1) $${Fe}^{2+}+HSO_{5}^{-}+H^{+}\to {Fe}^{3+}+{SO}_{4}^{\cdot -}+H_{2}O$$

(2) Fe2++HSO5−→Fe3++SO42−+O ·H+H+

(3) $${Fe}^{3+}+COF-COF\to {Fe}^{2+}+hole$$

(4) HSO5−+H2O→SO42−+3H++O ·2−

(5) O ·H+O ·2−+H+→O 12 +H2O

## 3. Materials and Methods

### 3.1. Chemicals and Materials

All reagents were purchased and used without further purification. 2,2′-bipyridine-5,5′-diamine (Bpy), 1,3,5-Tris (4-aminophenyl) benzene (TAB), and NaBH_3_CN were purchased from Bidepharm Co., Ltd. (Shanghai, China). 2,4,6-Triformylphloroglucinol (TP) and 2,5-dimethoxyterephthalaldehyde (DMTP) were purchased from Leyan Biopharmaceutical Co., Ltd. (Shanghai, China). Acetonitrile, acetic acid, anhydrous FeCl_2_, MB, Methyl Red (MR), Rhodamine 6G (Rh6G), and 2KHSO_5_·KHSO_4_·K_2_SO_4_ (PMS) were purchased from Aladdin (Shanghai, China).

### 3.2. Preparation and Characterization of Catalysts

#### 3.2.1. Preparation of Spherical DMTPCOF

First, a round-bottom flask was charged with TAB (281 mg, 0.8 mmol), DMTP (232 mg, 1.2 mmol), and acetonitrile (200 mL) was added, and the resulting suspension was sonicated for 5 min. Next, 5 mL of acetic acid was added to the mixture and stirred vigorously. Then, the reaction was allowed to stand at room temperature for 72 h. The obtained yellow precipitate was separated by filtration and washed with water and ethanol, then dried under vacuum for 6 h. DMTPCOF (432 mg) was obtained (89.9% yield).

#### 3.2.2. Preparation of Core-Shell DMTP-BpyCOF

A round-bottom flask was charged with Bpy (80 mg, 0.42 mmol), DMTPCOF (100 mg), and 100 mL of acetonitrile, and the mixture was sonicated for 30 min. Next, acetic acid (0.5 mL) was added dropwise, followed by sonication and stirring at room temperature for 2 h. Then, 20 mL of acetonitrile solution containing 2,4,6-Triformylphloroglucinol (60 mg, 0.28 mmol) was added, and the mixture was stirred for another 72 h. The brown precipitate was separated by filtration, washed with water and ethanol, and dried under vacuum for 6 h to obtain DMTP-BpyCOF (193 mg, 78.8% yield).

#### 3.2.3. Preparation of FeSAC@DMTP-BpyCOF

A round-bottom flask was charged with 100 mg of DMTP-BpyCOF, 1 mg of NaOH, 1 g of NaBH_3_CN, 25 mL of water, and 75 mL of ethanol, and the mixture was stirred for 8 h at −20 °C and then stirred for 24 h at room temperature. The brown precipitate was separated by filtration and washed with water and ethanol and dried under vacuum for 6 h. Finally, the obtained precipitate was charged into a round-bottom flask with 10 mg of FeCl_2_ and 50 mL of ethanol and stirred for 72 h at room temperature. The obtained brown precipitate was separated by filtration, washed with water and ethanol, and dried under vacuum for 6 h to obtain FeSAC@DMTP-BpyCOF (97 mg, 86.6% yield).

### 3.3. Measurements and Characterization

The chemical compositions of the prepared COFs were analyzed using X-Ray Diffraction (XRD, CuKα radiation, D8 Discover, Bruker (Saarbrucken, Germany)), Fourier-Transform Infrared Spectroscopy (FT-IR, TENSOR 27, Bruker (Saarbrucken, Germany)) at 400–4000 cm^−1^ and X-ray Photoelectron Spectroscopy (XPS, Escalab 250XI, Thermo (Waltham, MA, USA)). The morphologies were characterized using scanning electron microscopy (SEM, SU8010, HITACHI (Tokyo, Japan)), high-resolution transmission electron microscopy (HRTEM, FEI Talos F200X, Thermo Fisher (Waltham, MA, USA)), and specific surface area and porosity analyzer (BET, ASAP2460, Micromeritics). The thermal stability was analyzed using a Synchronous thermal analyzer (DTG, TA2000, Du Pont (Wilmington, DE, USA)). The degradation of dye was determined by a UV–visible spectrophotometer (UV-vis).

### 3.4. Degradation Experiments

The catalytic performance of FeSAC@DMTP-BpyCOF was evaluated by activating PMS for MB degradation. Typically, 1 mg of FeSAC@DMTP-BpyCOF and 0.5 mg of PMS were added to 10 mL of dye aqueous solution (50 mg/L). The degradation of MB was assessed by measuring the absorbance at 666 nm on a UV–visible spectrophotometer. The influence of concentrations of FeSAC@DMTP-BpyCOF, PMS, and dye, as well as the initial pH of the solution and the type of dye, were evaluated.

## 4. Conclusions

In summary, a ketoenamine single-atom iron catalyst FeSAC@DMTP-BpyCOF with core–shell structure was successfully constructed via sodium cyanoborohydride etching and iron atom coordination strategy. This study demonstrates that FeSAC@DMTP-BpyCOF exhibits superior efficiency in activating PMS for the degradation of MB. Moreover, the FeSAC@DMTP-BpyCOF catalyst exhibits excellent pH adaptability, versatility, and recyclability, which has potential applications for water pollution treatment. On the other hand, the surface reduction strategy for etching BpyCOFs expands the application range of ketoenamine COFs.

## Data Availability

The original contributions presented in the study are included in the article (and supplementary material), further inquiries can be directed to the corresponding authors.

## Supplementary Materials

The following supporting information can be downloaded at: https://www.mdpi.com/article/10.3390/molecules29153508/s1.
