# Supplementary material for: Single-Atom Iron Catalysts with Core-Shell Structure for Peroxymonosulfate Oxidation
Source: Molecules. 2024 Jul 26;29(15):3508. doi: 10.3390/molecules29153508 (PMC11313843; doi:10.3390/molecules29153508)
Supplement: Supplementary file 1 [file molecules-29-03508-s001.zip › molecules-3112816-supplementary.pdf]

Supporting Information

Single Atom Iron Catalysts With Core-Shell Structure for Peroxymonosulfate Oxidation

Jielei Fan, Ruoxue Wang, Xiaodong Zheng, Hancheng Jiang and Xiuli Hu*
Institute of Polymer Science and Engineering, School of Chemical Engineering, Hebei University of Technology, 300130, China.
* Corresponding author: Xiuli Hu; E-mail: huxiuli@hebut.edu.cn

Table S1. Degradation kinetic behaviour of different catalyst system

[Cat] g/L	$k_{absorption}$ min^{-1}	R^2	$k_{degradation}$ min^{-1}	R^2
0	/	/	0.006	0.9940
0.01	0.009	0.9663	0.016	0.9976
0.02	0.011	0.9665	0.025	0.9884
0.05	0.013	0.9694	0.038	0.9673
0.10	0.015	0.9973	0.138	0.9927
0.20	0.021	0.9774	0.165	0.9904

Table S2 Free radical quenching experiment's conditions

Quench item	Degrade system	Quencher	Degradation
Control group	[PMS] = 0.02 g/L, [Cat] = 0.1 g/L, [Mb]=0.05 g/L, 25℃, 60 min	/	90.74%
·OH and SO_4^{-}·	[PMS] = 0.02 g/L, [Cat] = 0.1 g/L, [Mb]=0.05 g/L, 25℃, 60 min	0.1 mM MeOH	75.61%
·OH	[PMS] = 0.02 g/L, [Cat] = 0.1 g/L, [Mb]=0.05 g/L, 25℃, 60 min	0.1 mM IPA	82.72%
$^1O_2$	[PMS] = 0.02 g/L, [Cat] = 0.1 g/L, [Mb]=0.05 g/L, 25℃, 60 min	0.01 mM BPQ	55.87%
O_2^{-}·	[PMS] = 0.02 g/L, [Cat] = 0.1 g/L, [Mb]=0.05 g/L, 25℃, 60 min	0.01 mM His	76.59%

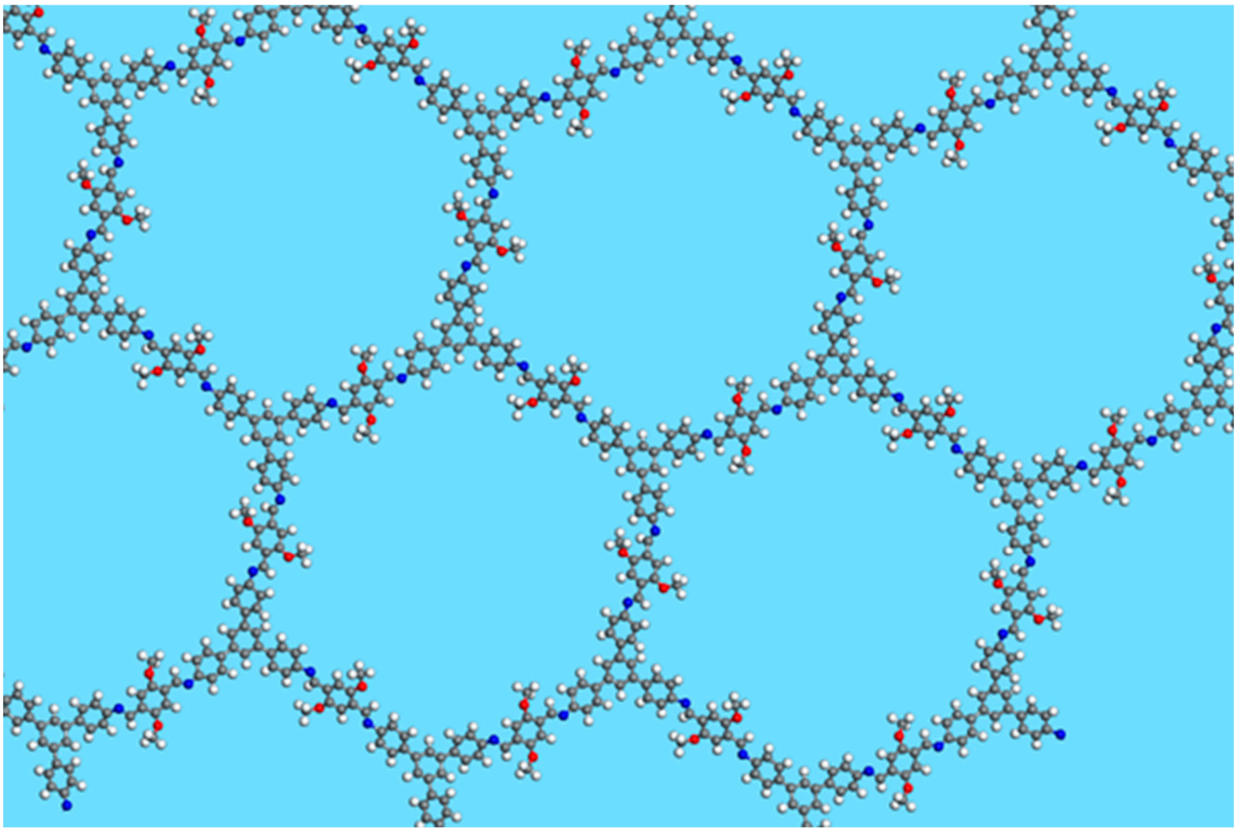

Figure S1. Crystal model of DMTPCOF

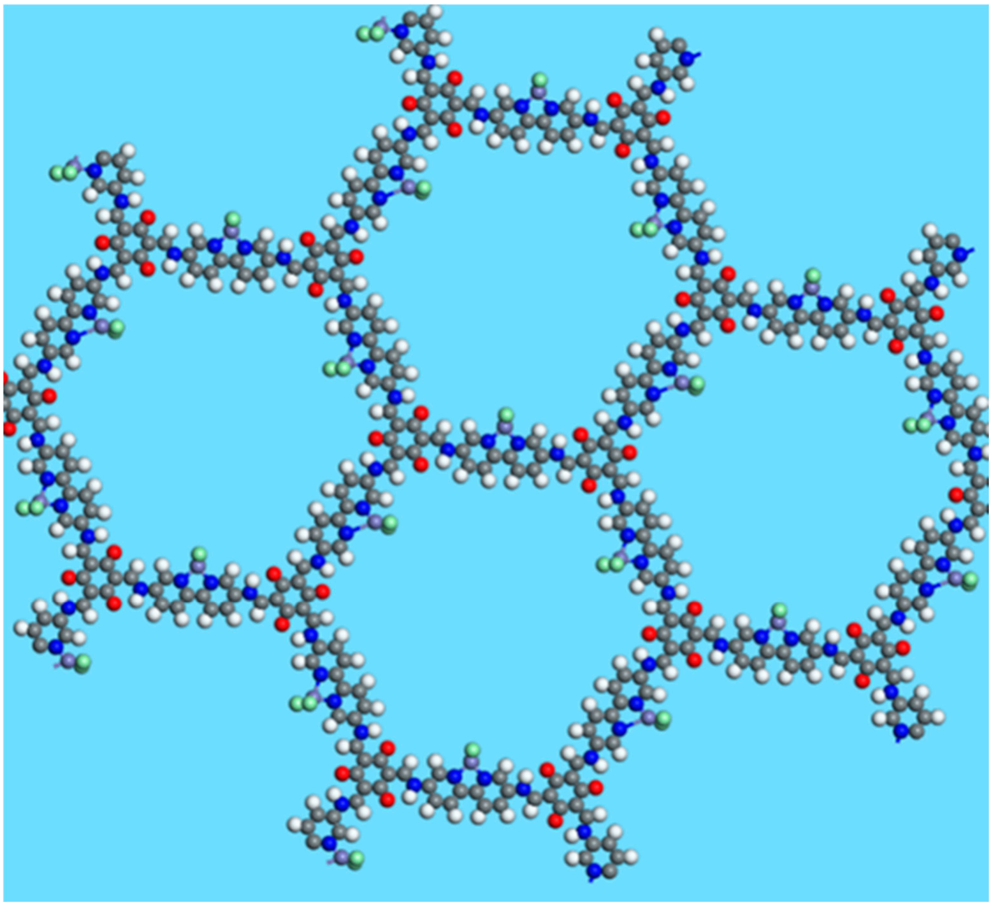

Figure S2. Crystal model of BpyCOF

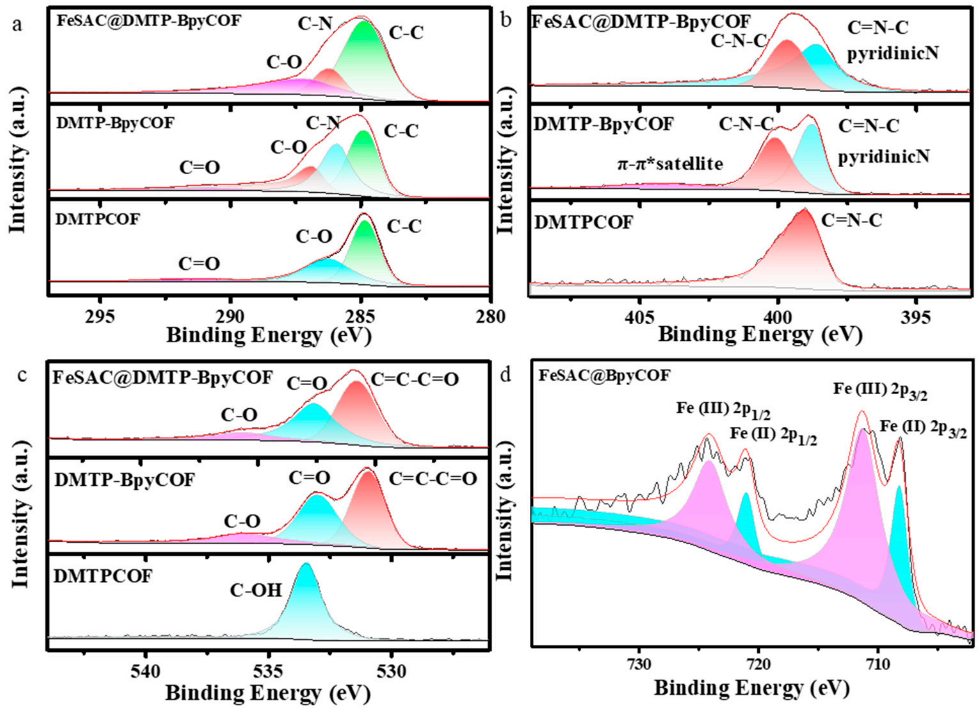

Figure S3. XPS profiles of FeSAC@DMTP-BpyCOF and its precursors (a) C1s (b) N1s (c) O1s (d) Fe2p

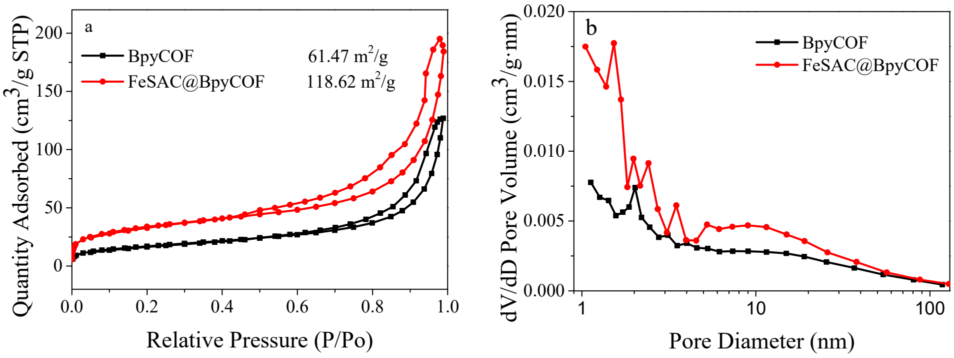

Figure S4 BET of FeSAC@BpyCOF and its precursors

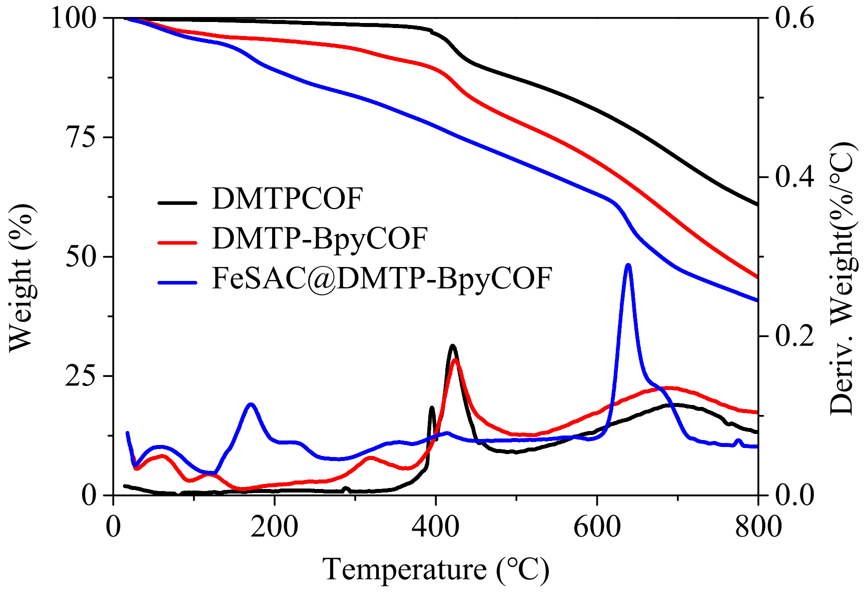

Figure S5. TG (left,up) and DTG (right,down) curves of FeSAC@DMTP-BpyCOF and its precursors

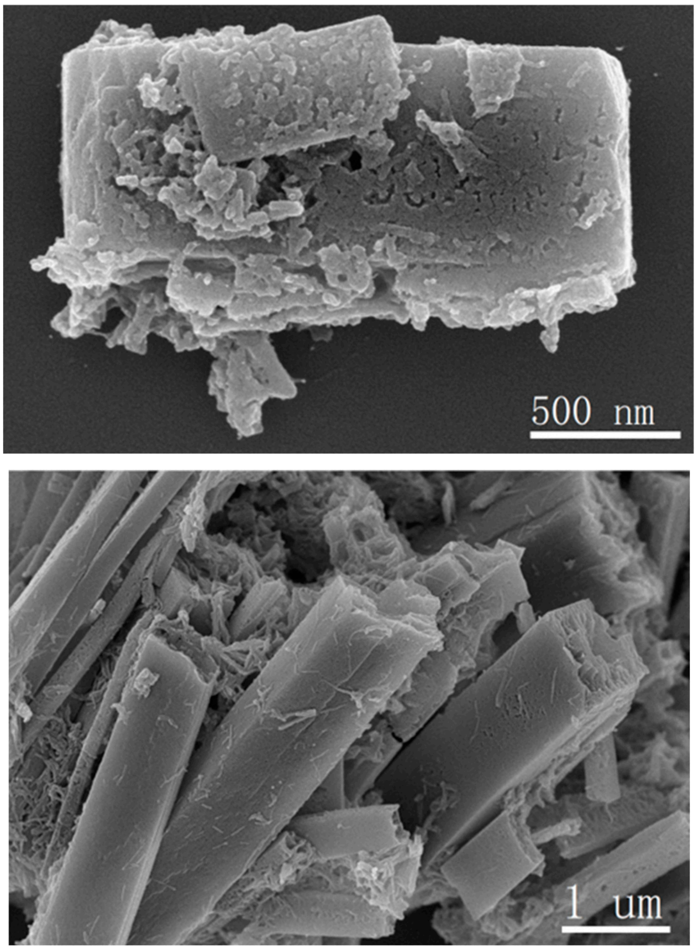

Figure S6. SEM images of BpyCOF

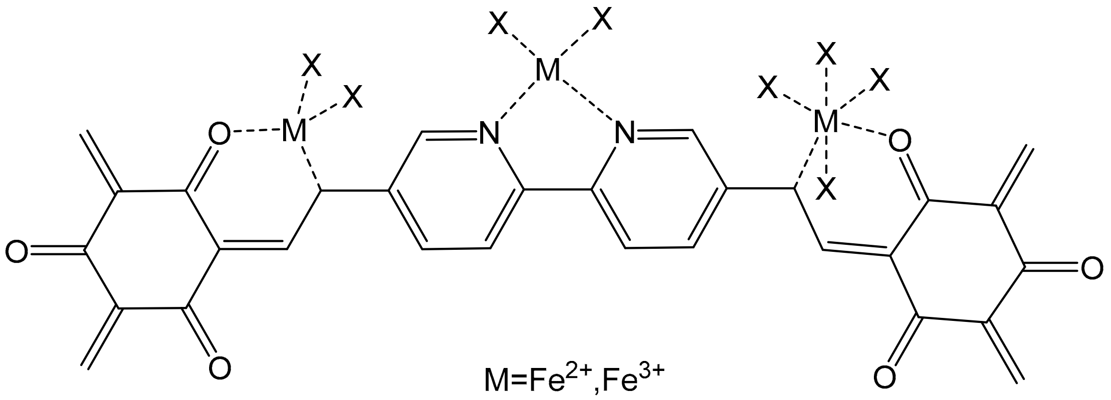

$M=Fe^{2+},Fe^{3+}$

$X=Cl^{-},CN^{-}$

Figure S7. The existence form and chemical environment of iron element

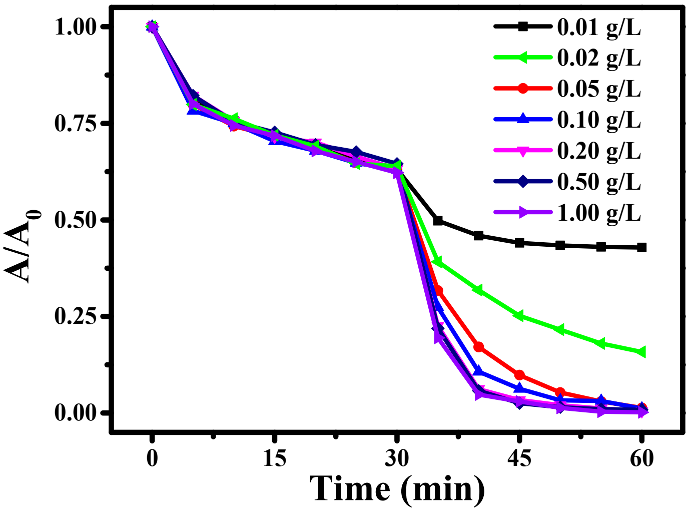

Figure S8. Effect of PMS concentration on the degradation of MB ([Cat] = 0.1 g/L, [MB] = 0.05 g/L)

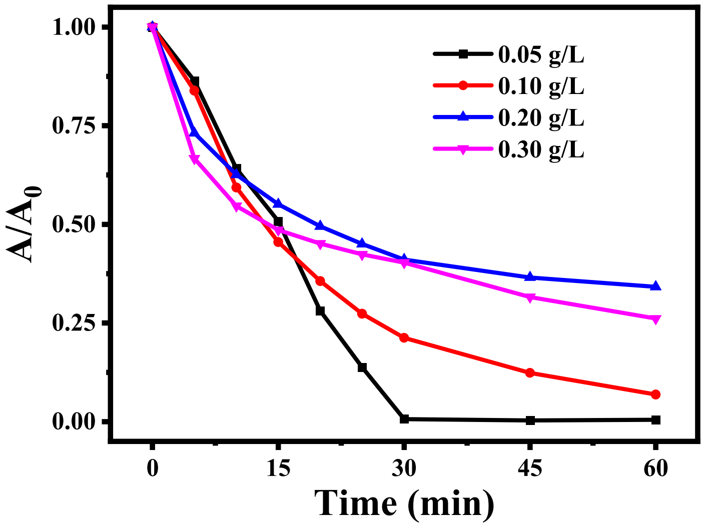

Figure S9. Effect of MB concentration on catalytic efficiency ([PMS] = 0.1 g/L, [Cat] = 0.1 g/L)

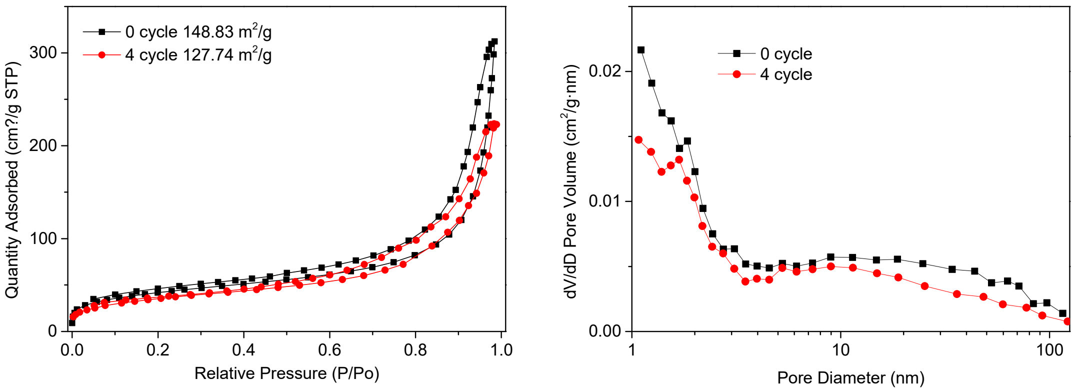

Figure S10. BET of FeSAC@DMTP-BpyCOF in cycle

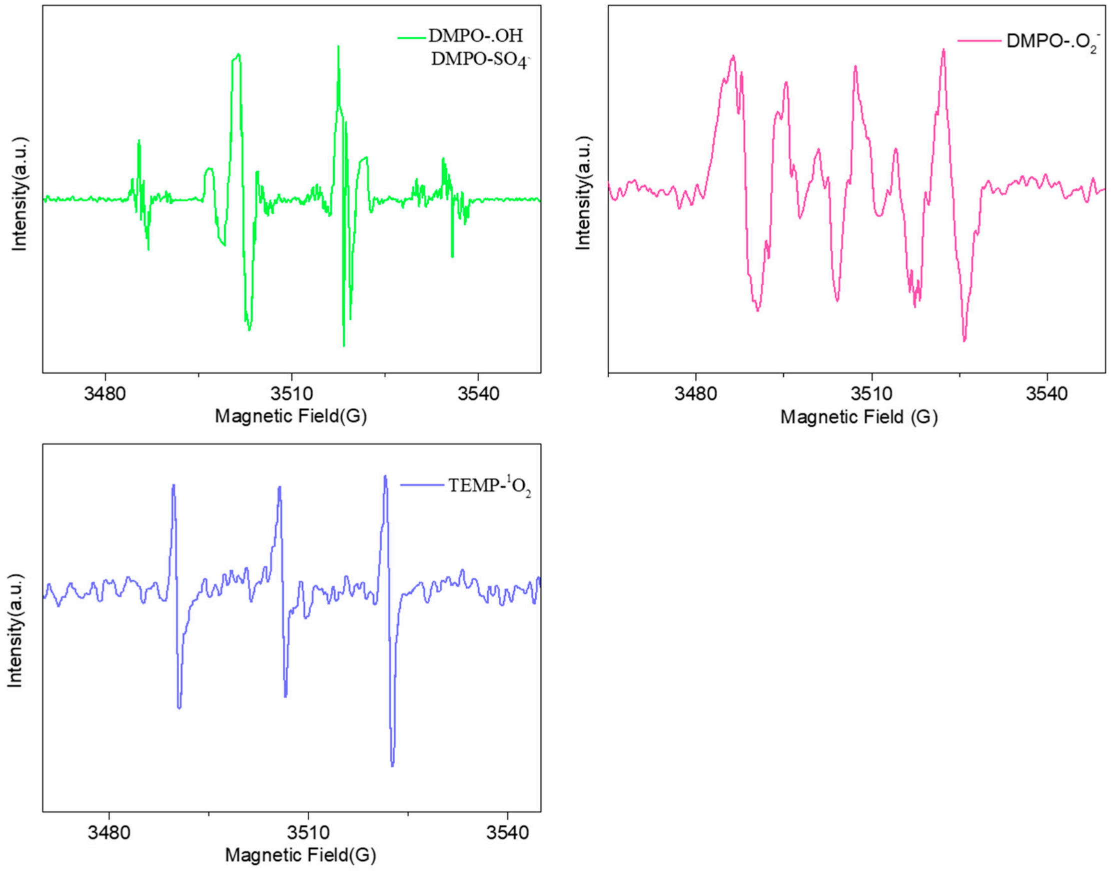

Figure S11. EPR spectra of the FeSAC@DMTP-BpyCOF/PMS system

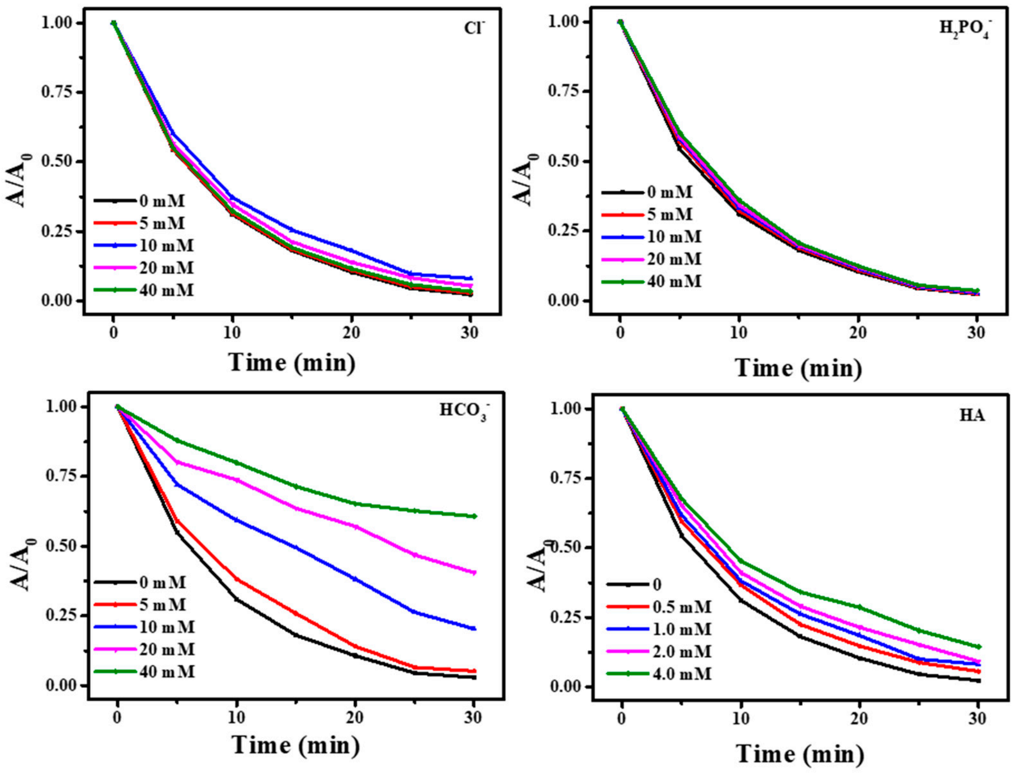

Figure S12. Influence of common ions and organics in water on degradation rates of MB([PMS] = 0.05 g/L, [Cat] = 0.1 g/L, [Mb] = 0.05 g/L)
